# Animal Bones Char Solubilization by Gel-Entrapped *Yarrowia lipolytica* on Glycerol-Based Media

**DOI:** 10.1100/2012/907143

**Published:** 2012-05-03

**Authors:** Maria Vassileva, Bettina Eichler-Lobermann, Antonia Reyes, Nikolay Vassilev

**Affiliations:** ^1^Department of Chemical Engineering, Faculty of Sciences, University of Granada, c/Fuentenueva s/n, 18071 Granada, Spain; ^2^Faculty of Agricultural and Environmental Sciences, University of Rostock, 18051 Rostock, Germany; ^3^Institute of Biotechnology, University of Granada, c/Fuentenueva s/n, 18071 Granada, Spain

## Abstract

Citric acid was produced with free and k-carrageenan-entrapped cells of the yeast *Yarrowia lipolytica* in single and repeated batch-shake-flask fermentations on glycerol-based media. Simultaneous solubilization of hydroxyapatite of animal bone origin (HABO) was tested in all experiments. The highest citric acid production by free yeast cells of 20.4 g/L and 18.7 g/L was reached after 96 h of fermentation in the absence and presence of 3 g/L HABO, respectively. The maximum values for the same parameter achieved by gel-entrapped cells in conditions of single batch and repeated-batch fermentation processes were 18.7 g/L and 28.1 g/L registered after 96 h and the 3d batch cycle, respectively. The highest citric acid productivity of 0.58 g L^−1^ h^−1^ was obtained with immobilized cells in repeated batch mode of fermentation when the added hydroxyapatite of 3 g/L was solubilized to 399 mg/L whereas the maximum efficiency of 89.0% was obtained with 1 g/L of HABO.

## 1. Introduction

The yeast *Yarrowia lipolytica *is one of the more intensively studied nonconventional yeast species and much progress has been made in areas related to its environmental and industrial applications in the past few years [1]. Organic acids, enzymes (proteases, lipases, esterases, and phosphatases), single-cell protein, and single-cell oil are the major products obtained from *Y. lipolytica*. The range of substrates used by *Y. lipolytica *include alkanes, fatty acids, organic acids, proteins, and some sugars, which also contribute for the interest in this yeast. Particularly, conversions of glycerol to value-added products including citric acid are of increasing interest due to the production of glycerol as a byproduct of biodiesel production [2].

 During the past few decades, numerous studies appeared limited mainly on registering yeasts in soil and the rhizosphere, without studying their functions and activities [3]. However, particularly the yeast *Yarrowia lipolytica* was widely experimented in studies on soil-plant systems due to its well-pronounced phosphate solubilizing activity [4], plant beneficial interactions with arbuscular mycorrhizal fungi and nitrogen-fixing bacteria [5, 6], and as a key element in soil restoration schemes [7].

In this work, yeast cells were employed in solubilization of a novel phosphate-bearing material, animal bone char, which is accepted as a waste derived from the meat industry. It should be noted that the current management of phosphate-bearing resources (mainly rock phosphates, RPs), which in fact are finite nonrenewable sources, is quite far from the principles of sustainability and may cause an escalating price increase mainly because of increases in RP-processing costs [8]. This situation determines the urgent need for alternative phosphate sources and clean biotechnological processes with high ecological effects [9]. A very attractive approach includes utilization of residues of combusted animal bones which are characterized by high calcium phosphate content. The aim of this work is to develop a biotechnological laboratory scheme based on microbial solubilization (*Y. lipolytica*) of a cheap, abundant, and heavy-metal-free P source (animal bone char) using a cheap and available industrial waste (glycerol).

## 2. Materials and Methods

### 2.1. Microorganism, Culture Maintenance, and Media Composition

The microorganism used throughout this study was *Yarrowia lipolytica*. The strain was maintained at 4°C on potato dextrose agar and subcultured every 3 months. The medium composition for free cell cultivation was as follows (g/L distilled water): glycerol, 60.0; (NH_4_)_2_SO_4_, 0.5; yeast extract, 1.0; K_2_HPO_4_, 1.5; MgSO_4_·7H_2_O, and 0.5; ZnSO_4_·7H_2_O, 0.007. Production medium for animal bone char solubilization by immobilized cells consisted of (g/L distilled water) glycerol, 100.0; yeast extract, 0.5; MgSO_4_·7H_2_O, and 0.2; ZnSO_4_·7H_2_O, 0.003. All chemicals were purchased from Sigma. Hydroxyapatite material (animal bone char, 20 mesh, 31% P_2_O_5_) was kindly provided by BES Ltd., Scotland. It was sterilized separately and added to the culture media before inoculation at a concentration of 1.0 to 4.0 g/L.

### 2.2. Production of Cell Mass for Immobilization

For the production of cell biomass, *Y. lipolytica* was cultivated in 300 mL Erlenmeyer flask containing 100 mL growth medium at 28°C on orbital shaker (agitation speed 150 rpm). After 40 h of fermentation, the yeast biomass was separated by centrifugation at 8000 g (20 min/4°C) and then washed twice with sterile distilled water. The wet cells obtained were used for further experiments with free cells and immobilization procedures.

### 2.3. Immobilization of Yeast Cells

20 g (wet weight) of yeast cells separated from the liquid culture broth was mixed with 100 mL 1 to 6% k-carrageenan (Sigma) solution. Carrageenan beads were formed in KCl (0.3 M, distilled water) by extrusion followed by hardening (20 min, gentle agitation) as described earlier [10]. Washed beads with sterile distilled water were used in further experiments.

### 2.4. Fermentation Processes

All experiments with free and immobilized cell cultivation for citric acid production and animal bone char solubilization were performed in triplicate in liquid submerged cultures (300 mL Erlenmeyer flasks containing 100 mL production medium). Before inoculation the flasks were sterilized at 121°C/20 min. Flasks were further inoculated with 0.5 g free or immobilized biomass. For the repeated-batch processes, the gel beads were separated from the medium, washed carefully with sterile distilled water, and further transferred into Erlenmeyer flasks with fresh production medium. In these experiments, after the immobilization procedure, gel-cell beads were cultivated for 48 h (except the single-batch experiment) and transfers were performed every 48 h. Fermentation processes were carried out at 28°C on orbital shaker with agitation speed of 200 rpm. In experiments with free cells without animal bone char addition pH was maintained with CaCO_3_ (3 g/L).

### 2.5. Analytical Methods

For biomass determination, 10 mL of culture was withdrawn from the flask and yeast cells were separated from the fermentation broth by centrifugation (8000 rpm, 20 min, 4°C), washed consecutively with 90% ethanol and twice with distilled water, and further dried at 80°C until constant weight. Glycerol and citric acid were determined in filtered (through 0.2 *μ*m pore size bacteriological filter, Whatman) aliquots of the culture by Enzytec Glycerol Test Kit and Enzytec Citric Acid Test Kit, respectively (N° E1224 and N° E 1214, R-Biopharm, Germany). Phosphate concentration in the fermentation broth was measured by the molybdo-vanado-method described by Lachica et al. [11].

## 3. Results and Discussion

### 3.1. Animal Bone Char Solubilization by Free Culture of *Yarrowia lipolytica*

Our first experiments were carried out with free cells of *Y. lipolytica* in order to test its ability to grow, produce, and release citric acid in the fermentation medium based on glycerol.

Briefly, the results of a single-batch submerged fermentation process of 120 h with free yeast cells showed very good growth on cultivation medium based on glycerol. After an intense growth during the first 48 h, when the biomass reached 8.8 g/L, the yeast culture entered a stationary phase, and at the end of the fermentation this parameter was fixed at 12.2 g/L. The amount of citric acid in the fermentation broth increased to the 4th day when it reached its highest value of 20.4 g/L and then decreased slightly to a final concentration of 18.1 g/L. The yield of citric acid based on the glycerol consumed varied from 0.47 g g^−1^ to 0.31 g g^−1^ registered in the beginning and the end of the fermentation process, respectively. The obtained data are similar to those reported by other authors on media containing glucose or glycerol as substrates [12–15]. However, it should be noted that the highest citric acid yield of 0.845 g g^−1^ reported in the literature was obtained on ethanol-based medium [16]. At the end of the studied period, an amount of 42 g glycerol per liter was found in the fermentation medium.

Bearing in mind the aforementioned results, another set of experiments was carried out to determine the ability of the free-cell yeast culture to solubilize animal bone char applied at 3.0 g/L. The presence of the phosphate material obviously affected the yeast metabolism as lower concentration of citric acid was detected in the fermentation broth (Table 1).

The maximum amount of citric acid of 18.7 g/L was 8.4% lower than that obtained in medium without animal bone char. In the same time, the biomass accumulated in the presence of animal bone char was 31.1% higher than that in experiments without the phosphate material. In these conditions, the yeast culture was capable of solubilizing the hydroxyapatite to soluble phosphate which reached its maximum of 331 mg/L after 96 h of fermentation. This concentration of soluble P corresponds to 35.6% of the total phosphate present in the culture medium. Although a repeated-batch process with free cells of *Y. lipolytica* is theoretically possible to perform, we did not carry out any experiment because of the difficulty of separating HABO from the yeast biomass. By this reason, in our further work (particularly in conditions of repeated-batch fermentations) it was not possible to compare results of citric acid production and animal bone char solubilization achieved with freely suspended cells.

### 3.2. Animal Bone Char Solubilization by Immobilized Cells of *Yarrowia lipolytica* in Conditions of a Single Batch Process

In the second part of the experiment, *Y. lipolytica* was entrapped in k-carrageenan and further employed in citric acid production and animal bone char solubilization. As a preliminary step, experiments aimed at selection of k-carrageenan concentration (1% to 6%) were carried out.

It was found that k-carrageenan concentration affected the citric acid production significantly. Yeast cells entrapped in gel beads formed by the lowest tested concentration of 1% were most effective as the quantity of citric acid between 11.0 g/L and 17.1 g/L achieved within first 72 h of the process was the highest amongst all treatments. However, this trend was followed by a sharp decrease in the citric acid production to 6.3 g/L at 96 h of the process. The highest level of citric acid production of 16.0 g/L was registered at 96 h with yeast cells immobilized in higher k-carrageenan concentration of 3%. At k-carrageenan concentrations higher than 3.0% the acid production decreased and reached the lowest level at experiments with 6% k-carrageenan. Based on the results obtained in this set of experiments, yeast cells entrapped in 3% k-carrageenan were selected for further experiments. Lower concentrations caused bead destruction and very strong release of free cell into the fermentation medium. Carrageenan concentrations higher than 3% obviously provoked mass transfer limitations that limit exchange of nutrients and metabolites within overall fermentation system.

In general, the results along the fermentation process were similar to those observed with free yeast cells. However, the maximum production of citric acid of 16.0 g/L and the corresponding productivity of 0.17 were lower than the respective values in the free-cell treatment. It should be mentioned that the yield (P/S) as measured along the entrapped-cell-based process was higher.

In our further work we tested the ability of the gel-entrapped yeast cells to solubilize the phosphate bearing material (Table 2). Results showed that the immobilized *Y. lipolytica* was at least as effective in solubilizing animal bone phosphate as its free form. The concentration of soluble phosphate in the fermentation medium reached 300 mg/L at the end of the process which was about 10.6% less than the 331 mg/L obtained with free cells. Another important fact in the single-batch experiments with immobilized cells is that the concentration of the residual glycerol of 53.8 g/L and 52.0 g/L at the end of the process were significantly higher than those measured with free cells. Similarly, yields of citric acid equal or higher than 50.0 g/g were calculated while in the last two experiments this parameter was 0.47 g/g as the highest.

The volumetric productivity Q_CA_ was stable within the range of 0.22–0.23 g L^−1^ h^−1^ which was equal that obtained in experiments with free cells and higher that obtained with immobilized cells without addition of bone char.

### 3.3. Solubilization of Animal Bone Char by Gel-Entrapped Yeast Cells Applied in a Repeated-Batch Fermentation Process

In conditions of repeated-batch fermentation process, the k-carrageenan-cell beads showed high efficiency at least during five repeated cycles (Table 3). The maximum level of soluble P in the culture broth, determined in the third batch cycle, was 399 mg per liter which was significantly higher than the maximum values found with free cells or immobilized cells in a single cycle.

The level of citric acid production was comparatively stable with a maximum of 28.1 g/L being reached at the 3d batch cycle. In conditions of repeated-batch fermentation, yields (P/S; g/g) between 0.52 and 0.60 were reached the later value being the highest amongst all treatments with free and immobilized cells of *Y. lipolytica*. Similarly, the citric acid process productivity of 0.44–0.58 g L^−1^ h^−1^ doubled that registered in previous experiments with free cells and immobilized cells.

### 3.4. Effect of Animal Bone Char Initial Concentration on P-Solubilizing Efficiency of Immobilized Cells of *Yarrowia lipolytica*

It should be mentioned that the highest concentration of soluble P obtained in repeated-batch process of 399 mg/L corresponds to 42.9% of the total P which was 7.3% higher than 35.6% of the total P present in the culture medium in experiments with free cells. Therefore, it seemed obligatory to test various initial concentrations of the P source to optimize the solubilization efficiency. The results from these experiments, carried out in a single 96-h fermentation process, can be seen in Table 4. The concentration of the soluble P varies from 269 mg/L to 341 mg/L, the latter being the highest value determined in a fermentation broth enriched with 4 g/L animal bone char. However, the phosphate solubilization efficiency in this case was the lowest one between all treatments.

The highest percentage (89.0) of soluble P calculated from the total in the applied phosphate material was reached in the treatment where 1 g animal bone char was used per liter. Therefore, when assessing the potential of the gel-entrapped *Y. lipolytica* we should compare the concentration of soluble phosphate and solubilization efficiency to select the best initial amount of animal bone char.

The results of this study could be evaluated in two directions. First of all, the ability of the yeast *Yarrowia lipolytica* to grow, produce citric acid, and solubilize insoluble phosphate on glycerol-based media was successfully proved. On the other hand, the use of animal bone char as a source of phosphate as a substitute of the traditionally applied rock phosphate was demonstrated.

Comparing to nonrenewable rock phosphates, animal bone char has a number of advantages which was recently reported by Vassilev et al. [8] It is also important to note that although composition of different rock phosphates varies depending on its type and origin, the majority of sedimentary rock-P deposits are rich in heavy metals which are not removed during production chemical processes [17] and further enter the food chain or contaminate soil and underground water. On the contrary, bone char is free from heavy metals while its P content is higher [18].

Bearing the above in mind, the present study could be further applied in production of cleaner fertilizer and/or introduction of gel-entrapped cells in plant-soil systems enriched with animal bone char.

## Figures and Tables

**Table 1:** Citric acid production and simultaneous animal bone char solubilization by free cells of *Yarrowia lipolytica*.

Time (h)	CA (g/L)	Biomass (g/L)	Res. g-ol (g/L)	Yield (P/S) (g/g)	Q_CA_ (g L^−1 ^h^−1^)	Soluble P (mg/L)
48	12.8 ± 0.1	10.1 ± 0.2	72.7 ± 1.1	0.47	0.27	176 ± 3.9
72	15.9 ± 0.4	13.9 ± 0.3	66.9 ± 2.1	0.40	0.22	232 ± 5.1
96	18.7 ± 0.8	14.2 ± 0.4	53.9 ± 1.5	0.37	0.19	331 ± 4.7
120	17.6 ± 0.5	16.0 ± 0.7	45.4 ± 1.8	0.42	0.15	307 ± 7.0

Values are means of ±SE of 3 replicates.

**Table 2:** Citric acid production and simultaneous animal bone char solubilization by k-carrageenan-entrapped cells of *Yarrowia lipolytica *employed in a single-batch fermentation process.

Time (h)	Citric acid (g/L)	Residual g-ol (g/L)	Yield (P/S) (g/g)	Q_CA_ (g L^−1^ h^−1^)	Soluble P (mg/L)
48	10.5 ± 0.3	78.8 ± 2.9	0.50	0.22	146 ± 4.9
72	16.8 ± 0.7	68.8 ± 2.0	0.54	0.23	231 ± 3.3
96	20.9 ± 0.6	58.9 ± 1.6	0.50	0.22	299 ± 11.1
120	18.3 ± 0.4	52.0 ± 2.2	0.40	0.15	301 ± 7.1

Values are means of ±SE of 3 replicates.

**Table 3:** Repeated batch citric acid production and simultaneous animal bone char solubilization by k-carrageenan-entrapped cells of *Yarrowia lipolytica *employed in a repeated-batch fermentation process.

Batch No.	CA (g/L)	Res. g-ol (g/L)	Yield (P/S) (g/g)	Q_CA_ (g L^−1^ h^−1^)	Soluble P (mg/L)
1	21.3 ± 0.8	58.8 ± 1.4	0.52	0.44	310 ± 9.2
2	25.6 ± 0.9	55.9 ± 2.1	0.57	0.53	368 ± 11.1
3	28.1 ± 1.0	51.9 ± 1.1	0.58	0.58	399 ± 8.8
4	26.7 ± 0.4	56.1 ± 2.0	0.60	0.56	375 ± 14.1

Values are means of ±SE of 3 replicates.

**Table 4:** Effect of animal bone char concentration on phosphate solubilizing efficiency of k-carrageenan-entrapped *Yarrowia lipolytica* in a 96-hour single-batch cycle.

HABO (g/L)	1.0	2.0	3.0	4.0
Soluble P (mg/L)	276 ± 22	269 ± 9	299 ± 12	341 ± 18
% P of total	89.0	43.4	32.2	27.5

Values are means of ±SE of 3 replicates.
